# Supplementary material for: Chromothripsis during telomere crisis is independent of NHEJ, and consistent with a replicative origin
Source: Genome Res. 2019 May;29(5):737–49. doi: 10.1101/gr.240705.118 (PMC6499312; doi:10.1101/gr.240705.118)
Supplement: Supplemental Material [file supp_gr.240705.118_Supplemental_file_1.zip › contigs/annotated_contigs/DB45/contig.2.DB45_length_422_mean_cov_9.22748815166.docx]

**DB45_length_422_mean_cov_9.22748815166**

```
TCCCCACCCCAACTACAGGGCCTGGGTCTCCGTGCGCCATCCCCCTCCCGCCCCCTCCCGCTCGCTCCCGCTGTTACAGGGCCCTAGGG
 >chr8:142425410-142425797 + E=6e-216 p=0e+00
ACCGCCCTGTCCCCCGTGGGCCCCAGAGCCCAGGCCAGCGCTGGAGGGACCCTCCTGCCCTCTGGCCGGGACCAGGGTGCCCGCCGCAG

CCCTGGGACCCTGCGGCCCCGGTCCCTATTCGAGGCCCCAGCCGGAGCGTTCCGGGATCCTTCGGGGGAGTGCCCAGCCCCGCGCCCCC

GCCTCCCACATGACTCCGGGTGGGCCCTTTTGACGTGCGCGGAGGGCGGGGGGCAGGGGGCGGGGCGGGGGGGCAGGGGGCAGGGGTGG

TGAAGGGGAGGGGGCGTGGCGGGGGGAGGGG|GAGGG|GGCACGGCGGGGAGAGGGGTAGGG|GCCGGG                                     >chr4:187403297-187403321 + E
```
